# Identifying spatially similar gene expression patterns in early stage fruit fly embryo images: binary feature versus invariant moment digital representations

**DOI:** 10.1186/1471-2105-5-202

**Published:** 2004-12-16

**Authors:** Rajalakshmi Gurunathan, Bernard Van Emden, Sethuraman Panchanathan, Sudhir Kumar

**Affiliations:** 1 Center for Evolutionary Functional Genomics, The Biodesign Institute, Arizona State University, Tempe, AZ 85287-5301, USA; 2 Department of Computer Science and Engineering, Arizona State University, Tempe, AZ 85287-8809, USA; 3 School of Life Sciences, Arizona State University, Tempe, AZ 85287-4501, USA

## Abstract

**Background:**

Modern developmental biology relies heavily on the analysis of embryonic gene expression patterns. Investigators manually inspect hundreds or thousands of expression patterns to identify those that are spatially similar and to ultimately infer potential gene interactions. However, the rapid accumulation of gene expression pattern data over the last two decades, facilitated by high-throughput techniques, has produced a need for the development of efficient approaches for direct comparison of images, rather than their textual descriptions, to identify spatially similar expression patterns.

**Results:**

The effectiveness of the Binary Feature Vector (BFV) and Invariant Moment Vector (IMV) based digital representations of the gene expression patterns in finding biologically meaningful patterns was compared for a small (226 images) and a large (1819 images) dataset. For each dataset, an ordered list of images, with respect to a query image, was generated to identify overlapping and similar gene expression patterns, in a manner comparable to what a developmental biologist might do. The results showed that the BFV representation consistently outperforms the IMV representation in finding biologically meaningful matches when spatial overlap of the gene expression pattern and the genes involved are considered. Furthermore, we explored the value of conducting image-content based searches in a dataset where individual expression components (or domains) of multi-domain expression patterns were also included separately. We found that this technique improves performance of both IMV and BFV based searches.

**Conclusions:**

We conclude that the BFV representation consistently produces a more extensive and better list of biologically useful patterns than the IMV representation. The high quality of results obtained scales well as the search database becomes larger, which encourages efforts to build automated image query and retrieval systems for spatial gene expression patterns.

## Background

The complexity of animal body form arises from a single fertilized egg cell in an odyssey of gene expression and regulation that controls the multiplication and differentiation of cells [1-3]. For over two decades, *Drosophila melanogaster *(the fruit fly) has been a canonical model animal for understanding this developmental process in the laboratory. The raw data from experiments consist of photographs (two dimensional images) of the *Drosophila *embryo showing a particular gene expression pattern revealed by a gene-specific probe in wildtype and mutant backgrounds. Manual, visual comparison of these spatial gene expressions is usually carried out to identify overlaps in gene expression and to infer interactions [4-6].

Whole fruit fly embryo and other related gene expression patterns have been published in a wide variety of research journals since late 1980's. These efforts have now entered a high-throughput phase with the systematic determination of patterns of gene expression [e.g., [7]]. As a result, the amount of data currently available has doubled leading to the imminent availability of multiple expression patterns of every gene in the *Drosophila *genome [7]. In addition, the use of micro-array technology to study *Drosophila *development has revealed additional and important insights into changes in gene expression levels over time and under different conditions at a genomic scale [8,9].

With this rapid increase in the amount of available primary gene expression images, searchable textual descriptions of images have become available [7,10,11]. However, a direct comparison of the gene expression patterns depicted in the images is also desirable to find biologically similar expression patterns, because textual descriptions (even using a highly structured and controlled vocabulary) cannot fully capture all aspects of an expression pattern. In fact, there is a need for automated identification of images containing overlapping or similar gene expression patterns [6,12] in order to assist researchers in the evaluation of similarity between a given expression pattern and all other existing (comparable) patterns in the same way that the BLAST [13] technique functions for DNA and protein sequences. Of course, unlike the genomes with four letters and proteomes with 20 letters, all gene expression anatomies cannot be easily reduced to, and thus represented by, a small number of components.

We previously proposed a binary coded bit stream pattern to represent gene expression pattern images [6]. In this digital representation, referred to as the Binary Feature Vector (BFV; BSV in [6]), the unstained pixels in the images (white regions and background) were denoted by a value of 0 and the stained areas (colored and foreground: gene expression) were denoted by a value of 1. Based on the BFV representations of the expression pattern, we proposed a Basic Expression Search Tool for Images (BESTi) [6] with an aim to produce biologically significant gene expression pattern matches using image content alone, without any reference to textual descriptions. We found that the BESTi approach generated biologically meaningful matches to query expression patterns [6].

In this paper, we explore how a more sophisticated Invariant Moment Vectors (IMV, [14]) based digital representation of gene expression patterns performs in generating an ordered list of best-matching images that contain similar/overlapping gene expression patterns to that depicted in a query image. IMV are frequently used in natural image processing (e.g., optical character recognition [15]) and have a number of desirable properties, including the compensation for variations of scale, translation, and rotation. If successful, IMV representations hold the promise of producing significantly shorter computing times for image-to-image matching compared to BFV.

Previously, we had examined the performance of the BFV representation for a limited dataset of early stage images [6]. Here we compare the relative performances of BFV and IMV first using a dataset containing 226 images (from 13 research papers). Then we test for scalability of the BESTi search by using a seven times larger dataset containing 1819 (1593 new + 226 previous) images from 262 additional research papers (list available upon request from the authors). Both datasets contained lateral views of early stage (1–8) embryos.

During these investigations, we also developed another measure of image-to-image similarity for the BFV representation. This measure is aimed at finding images that contain as much of the query image expression pattern as possible, but without penalizing for the presence of any expression outside the overlap region in the target image. In addition, we examined whether partitioning a multi-domain expression pattern into multiple BFV representations, each containing only one domain, yields a better result set.

Recently, Peng and Myers [16] have proposed a different procedure involving the global and local Gaussian Mixture Model (GMM) of the pixel intensities (of expression) to identify images with similar patterns. This GMM method is expected to find images with intensity and spatial similarities. This is different from the BFV and IMV methods examined here, which are intended to find only spatially similar patterns. This focus is important because, as mentioned in [6], the differences in gene expression intensity among images in published literature can arise simply due to use of different techniques, illumination conditions, or biological reasons. However, Peng and Myers method [16] appears to be promising and we plan to examine its effectiveness in a separate paper.

## Results and discussion

### Data set generation

An image database of 226 gene expression pattern images was initially generated using data from the literature [17-29]. All were lateral images and exhibited early stage (1–8) expression patterns. These images were selected because they had some commonality of gene expression (as seen by the human eye), which allowed us to evaluate the performance of the BESTi in finding correct as well as false matches under controlled conditions. BESTi was also tested for scalability on a larger dataset containing 1819 (1593 plus the 226) lateral views of early stage embryos. These 1593 images were obtained from 262 articles.

In order to present comprehensible result sets in this paper, we have primarily discussed the findings from the dataset of 226 and provided information on how those queries scaled when they were conducted for the larger dataset. In general, our focus was to show the retrieval of biologically significant matches based on both the visual overlap of the spatial gene expression pattern and the genes associated with the pattern retrieved.

Each image was standardized and the binary expression pattern extracted following the procedures described previously [6]. These extracted patterns, their invariant moments (*φ*_*1 *_through *φ*_*7*_), and binary feature representations were stored in a database. We also calculated and stored the expression area (the count of the number of 1's in the binary feature represented image), the X and Y coordinates of the centroid (, ), and the principal angle (*θ*) for each extracted pattern.

To quantify the similarity of gene expressions in two images, we computed two measures (*S*_S_, *S*_C_) based on the BFV representation (See equations 2 and 3 in **Methods**). *S*_*S *_is designed to find gene expression patterns with overall similarity to the query image, whereas *S*_*C *_is for finding images that contain as much of the query image expression pattern as possible without penalizing for the presence of any expression outside the overlap region in the target image. For a given pair of gene expression patterns (A and B), *S*_*S *_is the same irrespective of which image in the pair is the query image. That is, *S*_*S*_(A,B) = *S*_*S *_(B,A). This is not so for *S*_*C*_, because *S*_*C *_measures how much of the query gene expression pattern is contained in the image. Therefore, *S*_*C *_(A,B) ≠ *S*_*C*_(B,A).

For IMV representation, we computed one dissimilarity measure (*D*_*φ*_, equation 13 in **Methods**). Results from *D*_*φ *_should be compared to that from *S*_S_, as both of these measurements do not depend on the reference image, *i.e.*, *D*_*φ *_(A,B) = *D*_*φ*_(B,A) and, also they capture overall similarity or dissimilarity.

### Matches and their biological significance

The effectiveness of the BESTi in finding biologically similar expression patterns was geared towards determining the biological validity of the results obtained from the image matching procedure. All results were based solely on quantitative similarities between images without using any textual descriptions. All images were lateral views from the early stages of fruit fly embryogenesis and were oriented anterior end to the left and dorsal to the top. We refer to the images retrieved as the BESTi-matches.

#### Performance of BFV-*S*_*S *_search

**Figure 1:**
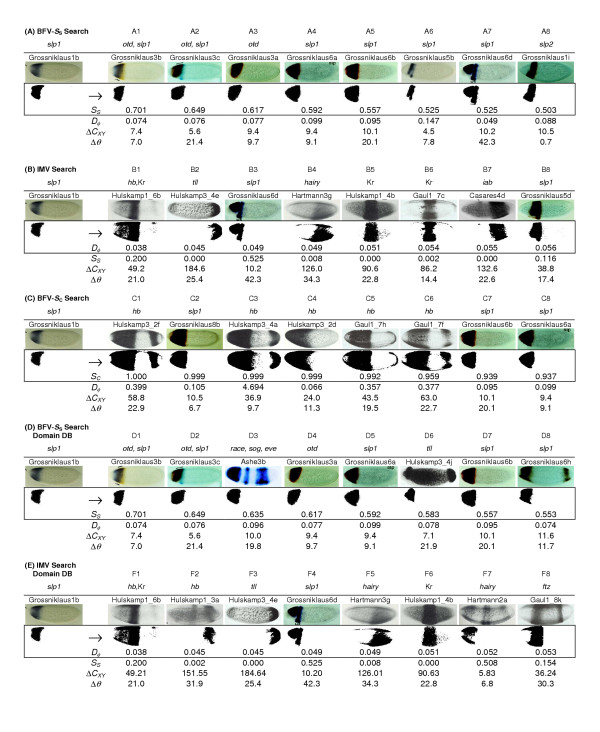
**BESTi search results with smaller dataset. **Results from the BESTi-search for the same query image [22] based on (A) BFV [*S*_*S*_], (B) IMV [*D*_*φ*_] and (C) BFV [*S*_*C*_] representations in the original dataset (226 images); and based on (D) BFV [*S*_*S*_] and (E) IMV [*D*_*φ*_] representations in the domain database (in which distinct domains of the multi-domain expression patterns were added to the original dataset as additional data points). The search argument and the results retrieved are shown on the left and right of the arrow, respectively. The original data used to generate these expression patterns are shown above this row. BESTi-matches are arranged in descending order starting with the best hit for the given search image. Values of difference in centroids (Δ*C*_*XY*_) and principal angles (Δ*θ*) are also given. Each image is identified by the last name of the first author of the original research article and the figure number with the following abbreviations: Ashe [19]; Casares [20]; Gaul1 [28]; Grossniklaus [22]; Hartmann [24]; Hulskamp1 [27]; Hulskamp3 [26].

Figure 1A shows the query image with gene expression restricted to the anterior (left) portion of the embryo, except that the expression is absent at the anterior terminus [22]. The query image depicts the expression of the *sloppy paired *(*slp1*) gene in a wildtype embryo. The BESTi-matches based on the *S*_*S *_measure for the representations are given in Figure 1A1–A8. BESTi retrieves images showing similar expression patterns, all of which are from same research article as the query image [22]. These images depict the expression patterns of *sloppy paired *genes (*slp1 *and *slp2*) in a variety of genetic backgrounds or in combination with a head gap gene *orthodentical *(*otd*); all of these genes are essential for the pattern formation in *Drosophila *head development [30]. In fact, *slp1 *and *slp2 *are tightly linked genes found in the *slp *locus of the *Drosophila *genome. They are not only closely related in their primary sequence structure, but also significantly similar in their expression pattern (compare Figure 1A7 and 1A8).

**Figure 2:**
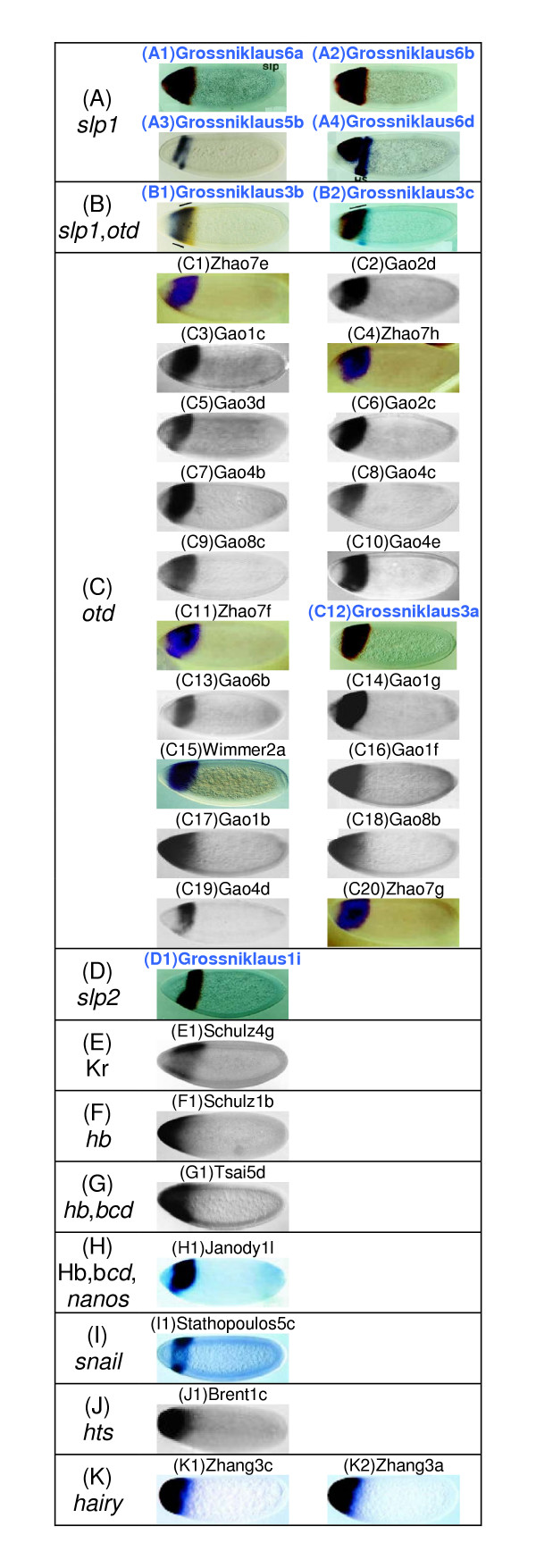
**BESTi search results for *S*_*S *_with larger dataset. **Comparison of search results from the small (226 images) and large (1819 images) dataset using the *S*_*S *_measure for the same query image (Figure 1A) [22]. Panels (A-K) are based on the genes whose expression patterns were retrieved as follows (A) *slp1*, (B) *slp1 *and *otd*, (C) *otd*, (D) *slp2*, (E) Kr, (F) *hb*, (G) *hb *and *bcd*, (H) Hb, *bcd *and *nanos*, (I) *snail*, (J) *hts *and (K) *hairy*. Images are referenced with the last name of the first author of the original article and its figure number: Grossniklaus [22]; Zhao [43]; Gao [44]; Wimmer [45]; Schulz1 [46]; Tsai [47]; Janody [48]; Stathopoulos [31]; Brent [32]; Zhang [33]. Common search results between the small and large dataset are indicated with dark blue image names.

A search was conducted using the same query image and same distance measure (*S*_*S*_) on the larger dataset. Figure 2 shows the top-35 matches, which contain all 8 matches shown in Figure 1A (images with blue colored legends). This allowed us to directly compare the quality of matches between the two datasets. Analysis of larger database of images yields more matches for the same *S*_*S *_cut-off value, as expected. A visual inspection reveals that these are all relevant images (Figure 2), with the larger dataset yielding more images for *otd *(20 images, Figure 2C). Images with expression patterns from *slp1*, *slp2 *and combined *otd *expression are found in Figure 2A,B, and 2D. More importantly, searches in the larger dataset provide images containing expression patterns of additional genes: *Kruppel *(Kr), *hunchback *(*hb*), *bicoid *(*bcd*), *nanos*, *snail*, *hu-li tai shao *(*hts*) and *hairy *(Figure 2E–K). Since these images did not exist in the smaller dataset, they were not included in the search results in Figure 1A. All are biologically useful matches because combinatorial input from gap genes (Kr, *hb*) along with *slp1 *establishes the domains of segment polarity genes in the head [22]. As for the *snail*, *hts *and *hairy *genes, there are no known interaction between them and *slp1 *(gene in the query image) in the wildtype embryo, but the images show overlap in gene expression due to the genetic backgrounds used [31-33]. Therefore, they are also biologically relevant matches.

#### Performance of IMV search

We used the same query image for the IMV method applied to the smaller dataset (*D*_*φ*_, results in Figure 1B) and compared the results to the BFV-*S*_*S *_search. In this case, we obtain images containing expressions of *hb*, Kr, *tailless *(*tll*), *slp1*, *hairy *and *infra-abdominal *(*iab*) (type I transcript). It is clear that IMV search produces some biologically disconnected matches. For example, Figures 1B2, 1B4–B7 exhibit no visual overlap in gene expression pattern with the query. Furthermore, even the biologically significant matches were retrieved out of order (Figure 1B1 before 1B3). This happens because *D*_*φ *_retrieves expression patterns that are of similar shape and/or size, regardless of the translation or rotation with respect to the query image.

**Figure 3:**
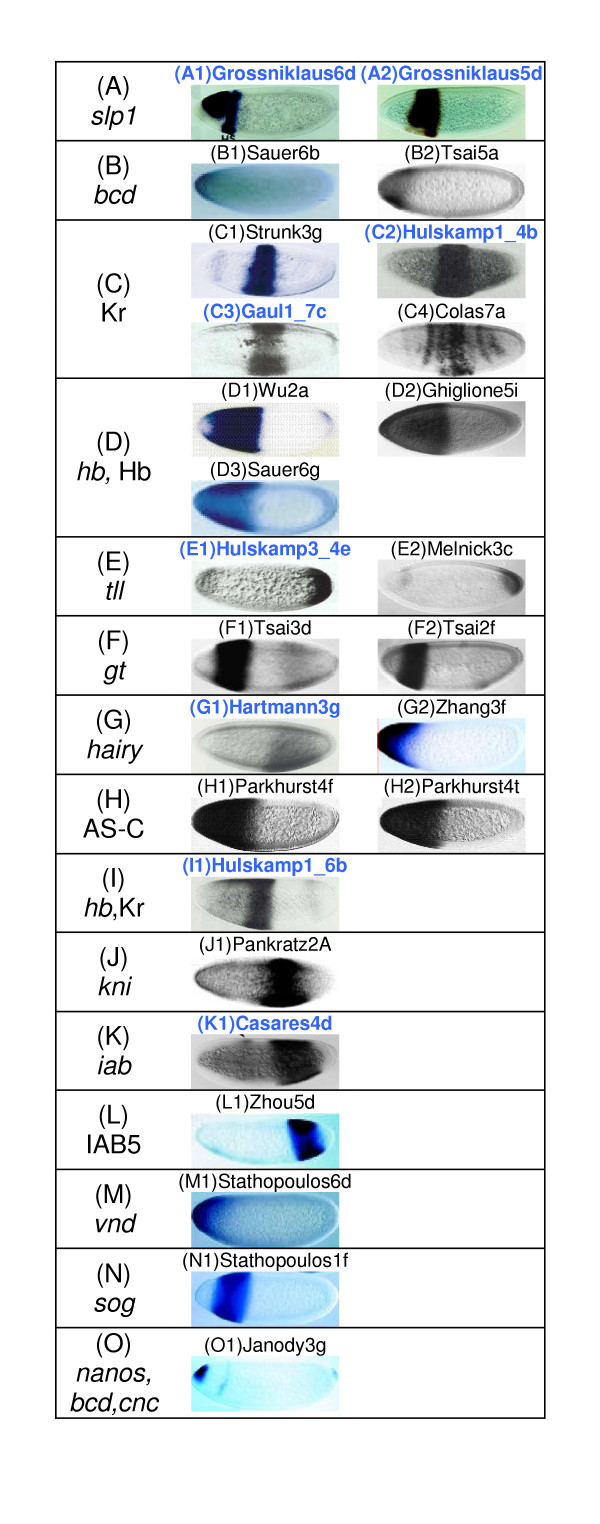
**BESTi search results for *D*_*φ *_with larger dataset. **Comparison of search results from the small (226 images) and large (1819 images) dataset using the *D*_*φ *_measure for the same query image (Figure 1A) [22]. Panels (A-O) are based on the genes whose expression patterns were retrieved as follows (A) *slp1*, (B) *bcd*, (C) Kr, (D) *hb*(D1,D3) and Hb(D2), (E) *tll*, (F) *gt*, (G) *hairy*, (H) AS-C, (I) *hb *and Kr, (J) *kni*, (K) *iab *(type I transcript), (L) IAB5 enhancer, (M) *vnd*, (N) *sog *and (O) *nanos*, *bcd *and *cnc*. Images are referenced with the last name of the first author of the original article and its figure number: Grossniklaus [22]; Sauer[49]; Tsai[47]; Hulskamp1[27]; Gaul1[28]; Strunk[50]; Colas[51]; Wu[52]; Ghiglione[53]; Pankratz[54]; Melnick[55]; Janody[48]; Zhang[33]; Parkhurst[56]; Zhou[57]; Stathopoulos[31]. Common search results between the small and large datasets are indicated with dark blue image names.

A comparison of the results from the smaller and larger dataset for the IMV measure is given in Figure 3. Twenty-six images were retrieved from the larger dataset when we used the same maximum distance value for the same query image. Of these, only two images were with expression pattern from *slp1 *(Figure 3 A1–A2). The expression of *bcd *was found in two of the results (Figures 3 B1–B2). 13 images containing gap gene expression patterns of Kr, *hb*, *tll*, *giant *(*gt*) and *knirps *(*kni*) (Figures 3 C1–C4, D1–D3, E1–E2, F1–F2, I1 and 3J) were also retrieved. Images with expression patterns of *hairy*, *achaete-scute *complex (AS-C), *iab *(type I transcript), IAB5 enhancer, *ventral nervous system defective *(*vnd*), *short gastrulation *(*sog*) and a combined expression of *bcd*, *nanos *and *cap 'n' collar *(*cnc*) accounted for the remaining nine (Figures 3 G1–G2, H1–H2, K1, L1, M1, N1 and 3O1). We see that the new results also suffer from the same problems as before. For example, images in Figure 3 C,E,K and 3L have no common expression pattern with the query image. Hence these are not biologically significant results even though few of them (Figures 3 C1–C4, E1–E2) contain expression patterns of developmentally connected genes (Kr and *tll *with *slp1*).

Since both *S*_*S *_and *D*_*φ *_measures capture the overall similarity or dissimilarity, we can use Figures 2 and 3 to compare the relative effectiveness of the BFV and IMV methods on the larger dataset. We clearly see that the BFV method performs much better in retrieving both overlapping and similar expression patterns that are also biologically significant.

In addition to the Hu moments, one could also compute Zernike moments, which are based on the polar coordinate system. Both Hu moments and Zernike moments are susceptible to the same problem namely expression patterns showing a similar shape but translated to different locations in the embryo would be in the same result set. We chose to study the Hu Invariant Moment Vectors mainly because the centroid of the image can be used to distinguish between similarly shaped but translated expression patterns. With Zernike moments, the image must be inherently contained within a unit circle anchored at the centroid [34]. Thus, there is no straightforward method to eliminate the translational problem.

Using the Hu moments, the spatial location problem can be corrected by considering the Euclidean difference in the centroid location expressed in pixels (Δ*C*_*XY*_) of the query and results. In the case of BFV-*S*_*S *_search results in Figure 1 (A1–A8), the maximum Δ*C*_*XY *_is less than or only slightly greater than the minimum Δ*C*_*XY *_for the IMV search results (Figure 1 B1–B8). Therefore, in the present case, the IMV-based BESTi search results need to be pared down using the centroid location difference. For example, if we consider results based on a Δ*C*_*XY *_lesser than or equal to 50 pixels, images shown in Figure 1 B2, B4–B7 would be removed producing a more meaningful result set.

#### Performance of BFV-*S*_*C *_search

Figure 1C shows the result for the same query image as used in Figure 1A, but using the newly devised *S*_*C *_distance for the BFV representation (BFV-*S*_*C *_search). This is expected to retrieve images with gene expression patterns that contain the largest amount of the overlap with the expression pattern in the query image. The top eight hits shown (Figure 1C1–C8) all contain over 93% of the query expression pattern: five of the matches are to the expression of *hunchback *(*hb*; C1, C3–C6) and the remaining three are from *slp1 *under different genetic backgrounds. As mentioned above, the combinatorial input from gap genes (including *hb*) along with *slp1 *establishes the domains of segment polarity genes in the head [22]. Therefore, gene expression patterns found by BFV-*S*_*C *_search are for developmentally connected genes. However, using the same query image, BFV-*S*_*C *_search yielded only two images in common with the BFV-*S*_*S *_results (Figure 1; C7 and C8 are the same as A5 and A4, respectively). This difference occurs because *S*_*S *_is designed to find gene expression patterns with overall similarity to the query image (Figure 1A), whereas *S*_*C *_is intended for finding images that contain as much of the query image expression pattern as possible and exclusive of the presence of the gene expression in the result image outside the region of overlap with the query image. Therefore, BFV-*S*_*S *_and BFV-*S*_*C *_have the capability of finding gene expression patterns from different biological perspectives.

**Figure 4:**
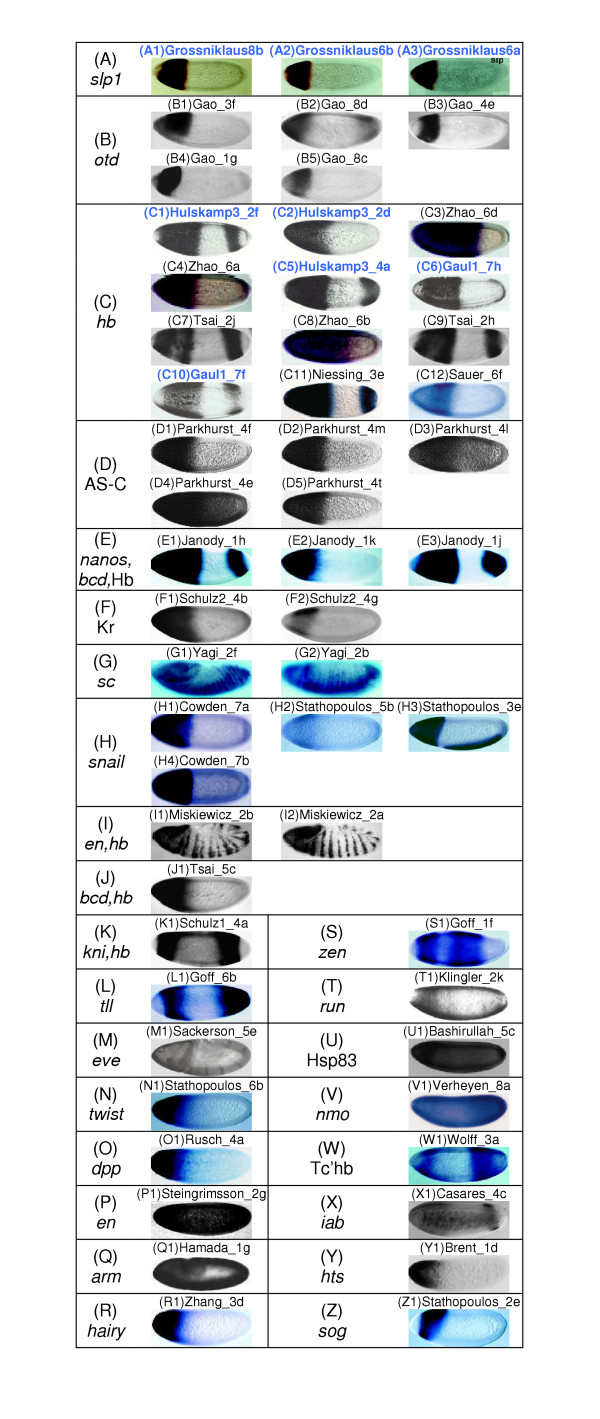
**BESTi search results for *S*_*C *_with larger dataset. **Comparison of search results from the small (226 images) and large (1819 images) dataset using the *D*_*φ *_measure for the same query image (Figure 1A) [22]. Panels (A-Z) are based on the genes whose expression patterns were retrieved as follows (A) *slp1*, (B) *otd*, (C) *hb*, (D) AS-C, (E) *nanos*, *bcd *and Hb, (F) Kr, (G) *sc*, (H) *snail*, (I) *en *and *hb*, (J) *bcd *and *hb*, (K) *kni *and *hb*, (L) *tll*, (M) *eve*, (N) *twist*, (O) *dpp*, (P) *en*, (Q) *arm*, (R) *hairy*, (S) *zen*, (T) *run*, (U) Hsp83, (V) *nmo*, (W) Tc'hb, (X) *iab*, (Y) *hts *and (Z) *sog*. Images are referenced with the last name of the first author of the original article and its figure number: Grossniklaus [22]; Gao [44]; Hulskamp1 [27]; Hulskamp3 [26]; Zhao [43]; Gaul1 [28]; Tsai [47]; Niessing [58]; Sauer [49]; Parkhurst [56]; Janody [48]; Schulz2 [46]; Yagi [59] Cowden [60]; Stathopoulos [31]; Miskiewicz [61]; Schulz1 [62]; Goff [63]; Sackerson [64]; Rusch [65]; Steingrimsson [66]; Hamada [67]; Zhang [33]; Klingler [68]; Bashirullah [69]; Verheyen [70]; Wolff [71]; Casares [20]; Brent [32]. Common search results between the small and large dataset are indicated with dark blue image names.

Using the same minimum similarity value for the BFV-*S*_*C *_in the larger dataset resulted in 55 images, given in Figure 4. Gene expression patterns of *slp1 *and *otd *accounted for 8 of these images (Figure 4A and 4B). 22 images contained expression patterns of the various gap genes *hb*, Kr, *kni *and *tll *(Figure 4C, 4E–F, 4I–L) that were co-expressed with *bcd *and *nanos *(Figure 4E and 4J) or with *en *(Figure 4I). Five other genes, developmentally connected to the gene, *slp1*, in the query image were also retrieved in this result set (*eve*, *twist*, *dpp *(*decapentaplegic*) [35]; *en *(*engrailed*) [36]; *arm *(*armadillo*) [37]; Figure 4M–Q). These images were not found in the top-35 of *S*_*S *_result set, which accentuates the different capabilities of the two BFV similarity measures in retrieving biologically relevant matches. The remaining images had expression patterns of AS-C, *sc *(s*cute*), *snail*, *hairy*, *zen *(*zerknullt*), *run*, Hsp83, *nmo *(*nemo*), Tc'hb, *iab*, *hts *and *sog *(Figure 4D, 4G–H, 4R–Z) which are not known to be directly related to the gene *slp1*. All but seven of these images (Figures 4 D3–D4, H1–H2, R1, X1 and 4Y1) were from a different developmental stage than the query image. Hence, by limiting the results to those from a specific stage, extraneous matches can be removed. The seven images having the same stage as the query image were retrieved because of their significant overlap (more than 94%) with the query gene expression pattern. Thus, we observe that the new distance measure *S*_*C *_has the potential to identify images containing expression patterns of developmentally connected genes, other than those retrieved by *S*_*S*_, thus improving the overall performance of the BFV method and the BESTi tool.

### Analysis of multi-domain gene expression patterns

Due to the presence of multiple areas of expression, some patterns in the database that appeared to contain much better matches (by eye and biologically) to the query image were not found or ranked very high. Hence, we also analyzed multi-domain expression patterns separately for the smaller dataset. Developmental biologists are also interested in finding such patterns as they contain overlaps with the expression domains in the query image. In fact, a large number of the expression patterns available today contain multiple isolated domains of expressions since more than one topologically distinct region of expression may be produced by many genes, transgenic constructs, probes or experimental techniques (multiple staining). In such cases, we need to consider each of these regions individually as well as in the context of the composite pattern. Biologically, it is important to consider them separately because different regions of expression may be under the control of distinct *cis*-regulatory sequences [e.g., [28,38]] or may represent the expression of different genes in a multiply-stained embryo.

Separating multi-domain gene expression patterns into individual components was straightforward; we simply generated multiple images from the same initial image and included them in the target dataset. This resulted in 192 additional images (418 total) in the database all of which were components of the initial gene expression patterns. The images were separated into expression regions horizontally and/or vertically depending on the gene expression. For this new set of images, the IMV as well as BFV representations were re-calculated and the BESTi query constructed as above.

Results from BFV-*S*_*S *_and IMV queries for this data set are given in Figures 1D and 1E, respectively. Now, many images with multiple regions of expression are retrieved in the result set (Figure 1D: D1–D8) and many of them show an even better match with the query pattern than those in Figure 1A for the BFV-based BESTi search. For instance, gene expression patterns are now retrieved (with more than 55% pattern similarity) from embryos with the expression of *tailless *(*tll*), which is known to interact with *slp1 *in defining the embryonic head [22], and with a composite expression of *race (related to angiotensin converting enzyme)*, *sog *(*short gastrulation*) and *eve *(*even-skipped*) due to enhanced *race *expression in the anterior domain caused by a transgenic construct causing ectopic expression of *sog *[19]. Therefore, the strategy of dividing multi-domain expression data into individual domains provides additional flexibility to query individual components or sub-sets of complex expression patterns. Results also improved for IMV (Figure 1E), but again the outcome reinforced the need to use the difference in centroid to limit the result set.

**Figure 5:**
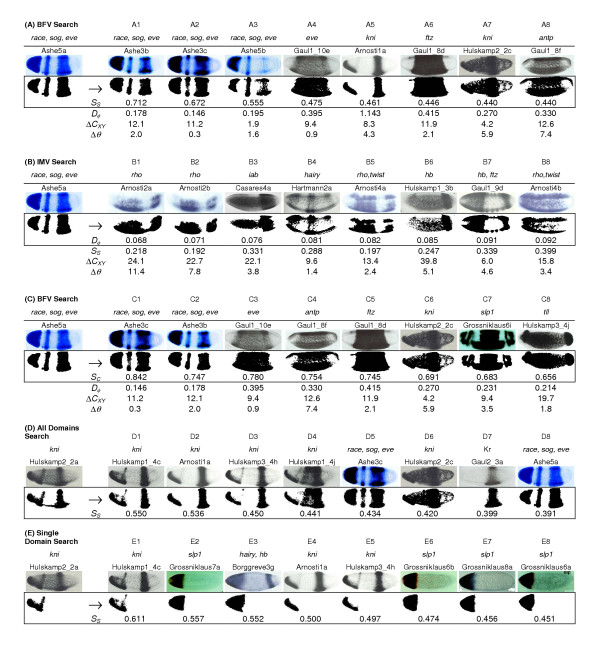
**BESTi search results with multiple domains of expression using smaller database. **Results from BESTi-search for a query image with multiple domains of expression. (A) BFV [*S*_*S*_], (B) IMV [*D*_*φ*_] and (C) BFV [*S*_*C*_] searches for the same expression pattern in the original database (226 images). (D) BFV [*S*_*S*_] search using the complete multi-domain expression in the original database and (E) BFV [*S*_*S*_] search using only the pattern on the left in the domain database. Search argument and the results retrieved are shown on the left and right of the arrow, respectively. Original data used to generate these expression patterns are shown above this row. BESTi-matches are arranged in descending order starting with the best hit for the given search statistic. Values of difference in centroids (Δ*C*_*XY*_) and principal angles (Δ*θ*) are also given for panels A, B and C. Each image is identified by the last name of the first author of the original research article and the figure number; with the abbreviations as follows: Ashe [19]; Arnosti [17]; Borggreve [18]; Casares [20]; Gaul1 [28]; Gaul2 [29]; Grossniklaus [22]; Hartmann [24]; Hulskamp1 [27]; Hulskamp2 [25]; Hulskamp3 [26].

Next we examine the performance of *S*_*S*_, *S*_*C *_and *D*_*φ *_in finding BESTi matches for a query pattern with multiple regions of expression (Figure 5A). This complex expression pattern consists of anterior and posterior domains caused by enhanced *race *expression resulting from dosage alteration of *dpp *in a *gastrulation defective *(*gd*) mutant background, and a middle stripe due to misexpressed *sog *using an *eve *stripe-2 enhancer [Figure 2d in [19]]. The results from this query are shown in Figure 5A1–A8 (only the original image set (226) was used as the target database in this case). We again find that *S*_*S *_finds many images from the same paper as well as some images from other research articles with similar expression patterns. The results correctly include expression pattern of *eve *(Figure 5A4), of another pair-rule gene (*ftz*: *fushi tarazu*; Figure 5A6), and of two other developmentally related genes [39,40].

When *D*_*φ *_is used as a search criterion, it produces some correct matches in the result set (Figure 5B1–B8). However, it generally fails to rank biologically meaningful matches as the best matches. Use of the centroid in this case is also not productive, as most of the matches show very close centroids. The principal angle (*θ*) value calculated does not show a significant difference in the early stage embryos used in this study. The results using the *S*_*C *_based search are given in Figure 5C1–C8. They show a number of images in common with the *S*_*S *_results. However, as expected, there are significant differences between the two searches.

The results in Figures 5D and 5E demonstrate the power of the BESTi-search when the multi-domain expression data are represented in their component patterns (domain database). In this case, all the BESTi searches are based on the use of *S*_*S *_as the search criterion. These searches are based on the complete expression (Figure 5D) and on one of its components (bottom-left domain, Figure 5E). All, but one, BESTi-matches in Figure 5D contain both domains of expression. In contrast, the use of only the left, anterior, domain (Figure 5E) in the BESTi search produces many other images in which the gene expression pattern is similar to only the anterior-ventral query pattern. Therefore, the use of individual expression components as search arguments increases the potential of directly identifying different overlapping expression patterns.

## Conclusions

We have found that it is possible to identify biologically significant gene expression patterns from a dataset by first extracting numeric signature descriptors and then using those descriptors in a computerized search of the database for expression patterns with similar signatures or maximum pattern similarities. We find that the BFV methodologies provide a longer and more biologically meaningful set of expression pattern matches than IMV. Even though IMV representations will produce much faster retrieval speeds for large collections of embryogenesis images, the lack of biological validity of BESTi-matches retrieved makes IMV undesirable for the present problem. Instead, investigations and strategies aimed at improving the real time performance of the BFV representation will better serve the developmental biological research.

## Methods

The wide variety of input methodologies, illumination conditions, equipment, and publication venues involved in the acquisition and presentation of gene expression patterns makes the available gene expression pattern data rather diverse. Extracting a gene expression pattern from its background requires the use of a combination of manual and automatic techniques. Each image is first standardized into a binary image as described in [6]. The standardized images are then represented using the Binary Feature Vector (BFV) [6], and the Invariant Moment Vectors (IMV) [14]. Similarity measures *S*_*S *_and *S*_*C *_are derived from BFV of which, *S*_*S *_is the one's complement of the distance metric *D*_*E *_presented in [6] and *S*_*C *_is a new measure introduced in this paper. The third metric *D*_*φ *_is deduced from the invariant moment vectors.

### Binary Sequence Vector analysis

The binary coded bit stream pattern, in which the two possible states indicate staining over or under a threshold value, is called as Binary Feature Vector (BFV). This is referred to as the Binary Sequence Vector (BSV) in [6]. In other words, we represent each image as a sequence of 1's and 0's, where the black pixels (stained areas) are denoted by a value of 1 and the white pixels (unstained and background) are denoted by a value of 0. This BFV holds the gene expression and localization pattern information of each image.

The expression patterns are ordered by evaluating a set of difference values, *D*_*E*_, between the binary feature vectors of every possible pair of images in the dataset. *D*_*E *_was introduced in [6] and is formally given as,

*D*_*E *_= *Count*(A XOR B)/*Count*(A OR B)     (1)

The term *Count*(A XOR B) corresponds to the number of pixels not spatially common to the two images and the term *Count*(A OR B) provides the normalizing factor, as it refers to the total number of stained pixels (expression area) depicted in either of the two images being compared. For simplicity, we use the one's complement of *D*_*E*_, as a measure of similarity of gene expression patterns between two images, *S*_*S*_, is given by the equation

*S*_*S *_= (1 - *D*_*E*_).     (2)

*S*_*S *_quantifies the amount of similarity based on the overlap between two expression patterns. *S*_*S *_is equal to 1 when the two expression patterns are identical (*D*_*E *_= 0).

We introduce a new similarity measure in this paper that does not penalize for any non-overlapping region. The measure *S*_*C *_quantifies the amount of similarity based on the containment of one expression pattern in the other given by

*S*_*C *_= *Count*(A AND B)/*Count *(A)     (3)

If the entire query image is contained within the result set images found in the database, *i.e.*, there is complete overlap (with respect to the query image) *S*_*C *_is equal to 1. Note that, *S*_*C*_(A,B) ≠ *S*_*C*_(B,A), because the denominator corresponds to the gene expression area of the query image.

### Invariant Moment Vector (IMV) analysis

Some methodologies of image analysis produce numeric descriptors that compensate for variations of scale, translation and rotation. In the following section, we describe the invariant moment analysis of gene expression data. Invariant moment calculations have been used in optical character recognition and other applications for many years [15].

To calculate these invariant moment descriptors the standardized binary image [6] is converted to a binary representation of the same pattern (BFV). From this binary sequence of the image, the invariant moments and other descriptors are extracted using the following method [14,41]. The continuous scale equation used is

*M*_*pq *_= ∬*x*^*p *^*y*^*q *^*f*(*x*, *y*)*dxdy*,     (4)

where *M*_*pq *_is the two-dimensional moment of the function of the gene expression pattern, *f*(*x*, *y*). The order of the moment is defined as (*p *+ *q*), where both *p *and *q *are positive natural numbers. When implemented in a digital or discrete form this equation becomes



We then normalize for image translation using  and  which are the coordinates of the center of gravity, centroid, of the area showing expression. They are calculated as



Discrete representations of the central moments are then defined as follows:



A further normalization for variations in scale can be implemented using the formula,



and  is the normalization factor. From the central moments, the following values are calculated:



where *φ*_*7 *_is a skew invariant to distinguish mirror images. In the above, *φ*_*1 *_and *φ*_*2 *_are second order moments and *φ*_*3 *_through *φ*_*7 *_are third order moments. *φ*_*1 *_(the sum of the second order moments) may be thought of as the "spread" of the gene expression pattern; whereas the square root of *φ*_*2 *_(the difference of the second order moments) may be interpreted as the "slenderness" of the pattern. Moments *φ*_*3 *_through *φ*_*7 *_do not have any direct physical meaning, but include the spatial frequencies and ranges of the image.

In order to provide a discriminator for image inversion (and rotation), sometimes called the "6", "9" problem, it has been suggested [14,42] that the principal angle be used to determine "which way is up". This is extremely important in embryo images because gene expression at the anterior and posterior regions may simply appear to be mirror images of each other to the invariant moments, but biologically they are completely distinct. The principal axis of the gene expression pattern *f*(*x*, *y*) is the angular displacement of the minimum rotational inertia line that passes through the centroid (, ) and is given as:



The slope of the principal axis is called the principal angle *θ*. It is calculated knowing that the moment of inertia of *f *around the line  is a line through (, ) with slope *θ*. We can find the *θ *value at which the momentum is minimum by differentiating this equation with respect to *θ *and setting the results equal to zero. This produces the following equation:



Using the condition |*θ*| < 45° one can distinguish the "6" from the "9" and rotationally similar gene expression patterns.

In invariant moment analysis, our initial method of image comparison calculates the Euclidean distance between the images using all moments (*φ*_*1 *_through *φ*_*7*_) and combinations of these moments. For example, if the first two invariant moments are used, then



and the distance *D*_*ij*_, between a pair of images *i *and *j *where *i*, *j *= 1, 2,...n is given by



This can be expanded to use all of the moment variables. Here, the Euclidean distance, *D*_*φ*_, between any two images is calculated as



where *i *and *q *designate images whose distance is being calculated and *j *designates the parameters used in the distance calculation and *j *= 1, 2, ..., 7. This assumes that all moments have the same dimensions or that they are dimensionless.

Using this method, it is possible to rank each of the images in order of their similarity based on, for example, the first two invariant moments that have clear-cut physical meanings. Expansion to include additional moments or parameters can be performed in a number of ways. It is possible to add additional parameters to the distance calculation making sure that each of the parameters has the same dimension. For example, *φ*_1 _has the dimension of distance squared, while *φ*_2 _has the dimension of the fourth power of distance, thus requiring the square root function to equalize dimensions for comparable distance calculation purposes. In general, the greater number of invariant moments used in the distance calculation, the more selective the ranking. We have also allowed for the use of the centroids and principal angle as a means of list limiting.

## Authors' contributions

SK originally conceived the project, developed the image distance measures based on the BFV representation, wrote an early version of the manuscript, and edited it until the final version. RG was responsible for writing new and using pre-existing programs to perform the image distance and parameter calculations, helped prepare the figures, searched the literature for gene expression data, maintained the database of gene expression pattern images, and helped in writing the manuscript. BVE provided the IMV method description, managed the day-to-day activities in the project, and did significant editing to produce the manuscript in the desired format for the journal. SP originally proposed the use of invariant moment vectors for biological image analysis, contributed significantly for the image distance and parameter calculations and provided critical feedback during the later stages of revision.
